# Predictive Factors of Dental Implant Failure: A Retrospective Study Using Decision Tree Regression

**DOI:** 10.7759/cureus.75192

**Published:** 2024-12-05

**Authors:** Sateesh G Shahapur, Kshitija Patil, Sakshi Manhas, Neetika Datta, Premraj Jadhav, Seema Gupta

**Affiliations:** 1 Department of Prosthodontics, Al-Ameen Dental College, Vijayapura, IND; 2 Department of Oral and Maxillofacial Surgery, Jawahar Medical Foundations Annasaheb Chudaman Patil Memorial Dental College, Dhule, IND; 3 Department of Periodontics, ITS Dental College, Greater Noida, IND; 4 Department of Prosthodontics, Clove Dental Clinics, Amritsar, IND; 5 Department of Prosthodontics and Crown and Bridge, Yogita Dental College and Hospital, Khed, IND; 6 Department of Orthodontics, Kothiwal Dental College and Research Centre, Moradabad, IND

**Keywords:** dental implant, failure, predictors, retrospective, survival analysis

## Abstract

Introduction: Dental implants are routinely used to replace missing teeth. Therefore, the primary aim of the present study was to assess the single-unit implant failure rate over a period of seven years from 2015 to 2021, with a minimum of two years post-implant follow-up. The secondary aim was to identify the risk factors associated with implant failure using machine learning decision tree regression and Kaplan-Meier survival analyses.

Materials and methods: An eight-year retrospective study was conducted using the clinical records of 224 patients who received single-unit dental implants between January 2014 and December 2021, where risk factors for early (EIF) and late implant failure (LIF) were identified. The patients’ clinical case records and radiographs were used to assess implant failure.

Results: Smoking and peri-implantitis were principal contributors to failure (p=0.001). Implant failure was more common in males, the maxillary jaw, and posterior teeth, although these factors were not significantly associated with implant failure (p>0.05). The duration of failure was 16.87±4.6 months for LIF, in contrast to 5.71±1.38 months in EIF. Bruxism and peri-implantitis were correlated with diminished survival duration, especially when compounded by additional risk factors such as diabetes mellitus. Isolated peri-implantitis yielded an average failure duration of approximately 13.4 months, whereas bruxism intensified the failure interval to approximately 13.8 months. Kaplan-Meier survival analysis revealed that among the identified causes of failure, peri-implantitis and smoking were the predominant factors, followed by bruxism, diabetes, and complications related to osseointegration.

Conclusion: Age, sex, type of surgical procedure, sinus lift, and grafting procedures were not significantly associated with dental implant failure, whereas bruxism, peri-implantitis, lack of osseointegration, smoking, and type 2 diabetes mellitus were significant predictors.

## Introduction

Endosseous dental implants have significantly transformed the restoration options available for individuals who are either completely or partially edentulous. The elevated survival rates documented for the replacement of a single tooth have underscored the efficacy of implant-supported restorations as a viable strategy for oral rehabilitation [1]. A comprehensive systematic review conducted by Hjalmarsson et al. encompassed nine investigations in which 367 individuals with solitary implants were monitored for a decade following implant placement [2]. According to both patient- and implant-level data, the survival rates for the implants were 93.8% and 95.0%, respectively. The survival rate of original crown restorations was 89.5%. Many studies have reported high survival rates of dental implants ranging from 93% to 100% for single-tooth replacement [3,4].

Qian et al. reported that the 10-year cumulative survival rate was 90.7% in cases where the sinus lift procedure was performed along with bone grafting and 95.0% in cases where the sinus lift procedure was performed without bone grafting [5]. Ramalingam et al. reported success rates of 97.3% for mandibular implants and 94.9% for maxillary implants [6]. Implants of 4.3 x 8 mm and 3.5 x 10 mm were the least successful (91.7%). Dental implant failure is categorized as an early or late implant failure. Early implant failure refers to an implant exhibiting clinical mobility before the installation of a definitive prosthesis. This phenomenon typically arises due to biological complications whereby the organism fails to integrate the implant, often referred to as "rejection" of the dental implant. The factors contributing to early implant failure may involve immunological, genetic, and environmental factors. Conversely, late implant failure transpires within one to three years after implantation [7].

Infection is the predominant preventable factor contributing to the failure of dental implants. At any stage during the course of implant treatment, the onset of bacterial infection can lead to implant failure. Peri-implantitis refers to an inflammatory reaction characterized by bone resorption in the soft tissues surrounding the implants. Peri-implantitis may encompass infections induced by plaque accumulation on the exposed surfaces of biomaterials [8]. Therefore, the primary aim of the present study was to assess the single-unit implant failure rate over a period of seven years from 2015 to 2021, with a minimum of two years post-implant follow-up. The secondary aim was to identify the risk factors associated with implant failure using decision tree regression and Kaplan-Meier survival analyses.

## Materials and methods

Study design and setting

**Figure 1:**
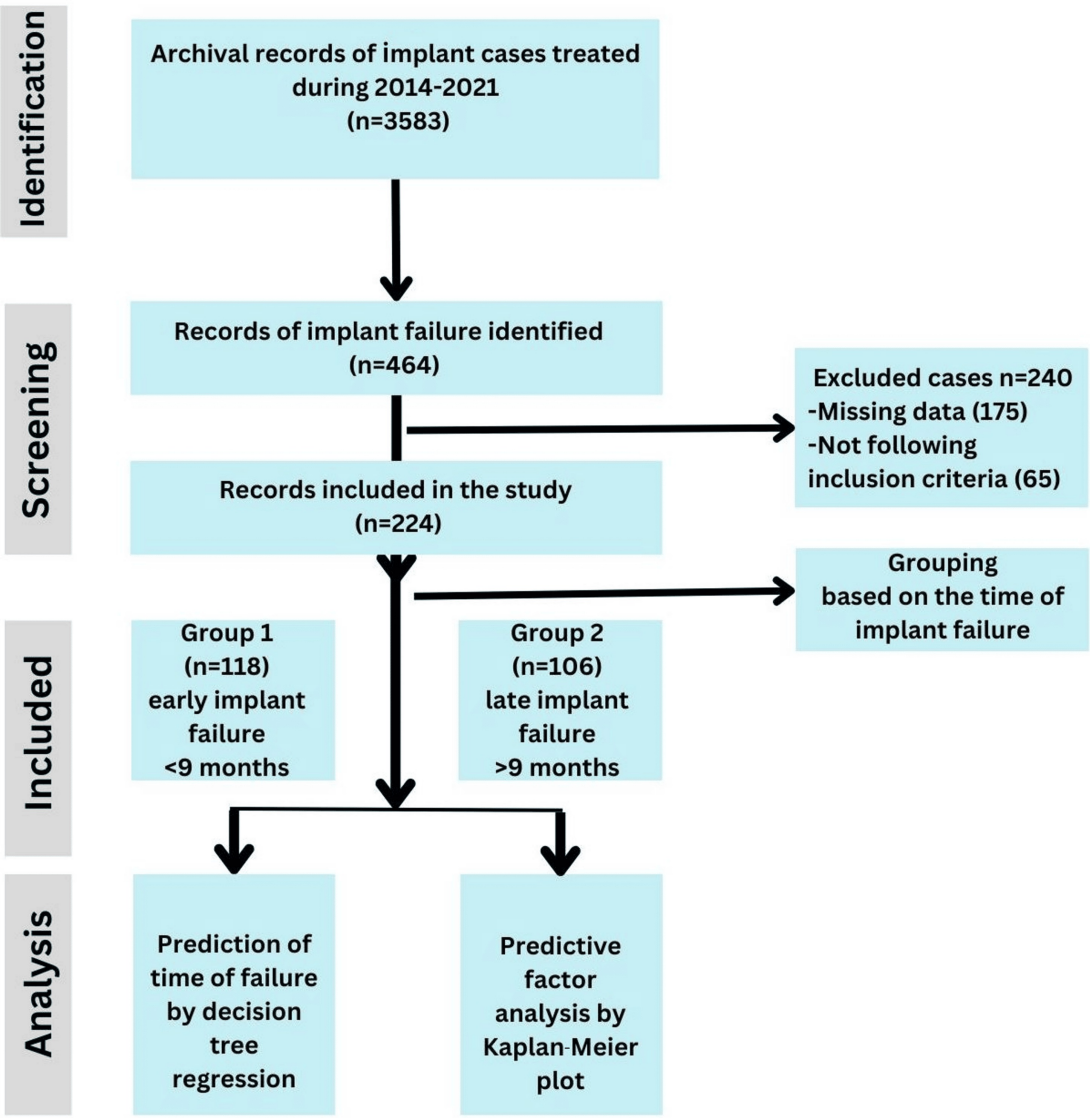
Study design according to STROBE guidelines. STROBE: Strengthening the Reporting of Observational Studies in Epidemiology

This retrospective, observational cohort study was conducted using the records of patients who visited the Department of Prosthodontics, Yogita Dental College, Khed, between January 2014 and December 2021. The study was conducted in accordance with the Strengthening the Reporting of Observational Studies in Epidemiology (STROBE) guidelines and principles of the Declaration of Helsinki. Ethical committee approval was obtained (#EC/NEW/INST/2023/2512). As a routine protocol, written consent is always obtained from all patients to use their records for research purposes while maintaining confidentiality (Figure 1).

Sample size estimation

To ensure adequate statistical power, the required sample size for this study was estimated based on an 80% confidence level, a 5% margin of error, and a previously reported dental implant failure rate of 5.7% [9]. The sample size calculation used the following standard formula for estimating proportions: n=Z^2^×p×(1−p)/E^2^, where n is the required sample size, Z is the z-score for the desired confidence level (1.28 for 80% confidence), p is the prevalence rate (0.057), and E is the margin of error (0.05). Applying these values, the estimated sample size required was approximately 204 participants.

Eligibility criteria

In the current study, implant failure was characterized by increased implant mobility, pain, and infection. “Lost implants” were also considered as failed implants. The inclusion criteria included individuals of diverse age groups and both sexes who experienced implant failure subsequent to the placement of a minimum single-unit dental implant, adhering to the established contraindications for implantation. Before the surgical intervention for implant placement, dental care and periodontal management were provided by the treating clinician. All participants underwent a periodontal maintenance regimen both before and after the surgical procedure. All cases were treated following sterilization protocols and standard procedures. Patients with a single-unit implant success and those who did not complete the minimum follow-up of two years were excluded. The successful outcome of an implant was characterized as being “operational, accepted, and not necessitating removal.”

Methodology

A total of 224 patients were included in the study. Cases of failed single-unit implants were further classified as early or late implant failure. Early implant failure (EIF) is characterized by significant implant mobility and/or the presence of pain or infection, which may include peri-implant radiolucency, occurring within a timeframe of six months post-implantation. Furthermore, implant loss during this interval was categorized as EIF. In contrast, the emergence of pathological radiological or clinical features, along with the loss of an implant commencing after a latency period of six months, was classified as late implant failure (LIF). The patients’ records were screened for basic details such as age, sex, extraction history in case of missing teeth, detailed medical history, history of tobacco use (smoking or smokeless), alcohol consumption, oral hygiene status as evaluated by bleeding index, probing depth, immediate or delayed loading protocol, type of implant placed, implant length and diameter, pre-implant bone surgery if required, site of placement, and presence of parafunctional habits such as bruxism and clenching.

Biological complications included implants with peri-implantitis (progressive marginal bone loss of at least 3 mm, with a probing depth >6 mm). Biomechanical complications include (screw loosening, debonding, prosthesis delamination or fracture, and abutment or implant fracture) [10]. The technical procedures were evaluated by two experienced clinicians. Cone-beam computed tomography (CBCT) scans were obtained as a routine procedure for pre-planning records for implant placement in all patients. Routine follow-ups were conducted for all patients at intervals of six months. Frequent follow-ups were scheduled for all cases in which signs of pain, inflammation, or delayed healing were observed. Intraoral periapical radiographs were obtained at follow-up visits, as required.

Surgical procedure

The cases chosen for this study had all implants inserted under sterile conditions in accordance with the manufacturer's specifications. Prior to the preparation of the implantation site, augmentation methods were employed if required based on the individual characteristics of the patients such as direct or indirect sinus lift with or without bone grafting. Non-salvageable teeth were extracted, allowing bone healing for eight to 16 weeks. The protocol was implemented in accordance with the patient's overall health, clinical circumstances, and quality and quantity of bone present. All the implants were placed using a surgical guide, and after raising the flap by a single clinician with more than 10 years of experience (PJ). The clinician determined the immediate or delayed loading based on the specific characteristics of the patients. In instances of delayed loading, prosthetic loading was performed in adherence to surgical protocols (including hygiene, accuracy, and management of soft tissue) following a latency period of either three to four months for implants placed in the mandible or four to six months for those inserted in the maxilla. Care was taken to thoroughly verify both static and dynamic occlusions.

Statistical analysis

The acquired data were inputted into Microsoft Excel for statistical evaluation using Jeffreys’ Amazing Statistics Program version 0.19.0, JASP 2024 (Amsterdam, Netherlands: JASP Team). Normality was assessed using the Shapiro-Wilk test, which revealed that the data conformed to the assumptions of normality. Categorical variables were represented as frequencies and percentages, while proportional distributions were analyzed using the chi-square test. Continuous variables were expressed as mean and standard deviation (SD). A t-test was performed to compare means across various groups. Additionally, a machine learning model was established for decision tree regression analysis aimed at predicting the duration (in months) until the failure of dental implants. The independent factors contributing to implant failure were examined using Kaplan-Meier analysis.

## Results

The investigation indicated that cases of implant failure were mostly observed in males than females, although the difference was not statistically significant between the EIF and LIF groups (p=0.992). EIF and LIF were mainly observed in the maxillary jaw and posterior region. Seventy six (34%) patients had diabetes, and 54 (24%) were smokers. Smoking and diabetes had a statistically significant effect on implant failure, with smokers exhibiting elevated EIF in 36 (16.07%) cases, and diabetic individuals demonstrating increased LIF in 46 (20.54%) cases. Grafting was required in 86 (38%) cases, immediate implant placement in 70 (31%) cases, and delayed implant placement in 154 (68%) cases. The principal contributors to failure were smoking and peri-implantitis (p=0.001) (Table 1).

**Table 1:** Association of various factors with early and late implant failure. *P-value <0.05 was considered significant. Data were presented in the form of n (%).

Parameters	Category	Late failure, n (%)	Early failure, n (%)	Chi-square value	p-Value
		106 (47%)	118 (53%)		
Gender	Male	70 (31.25)	78 (34.82)	0.01	0.992
	Female	36 (16.07)	40 (17.86)		
Jaw	Maxilla	66 (29.46)	86 (38.39)	2.89	0.089
	Mandible	40 (17.86)	32 (14.29)		
Site	Anterior	30 (13.39)	46 (20.54)	2.84	0.092
	Posterior	76 (33.93)	72 (32.14)		
Smoking	No	88 (39.29)	82 (36.61)	5.58	0.018*
	Yes	18 (8.04)	36 (16.07)		
Diabetes	No	60 (26.79)	88 (39.29)	8.05	0.005*
	Yes	46 (20.54)	30 (13.39)		
Graft required	No	64 (28.57)	74 (33.04)	0.13	0.721
	Yes	42 (18.75)	44 (19.64)		
Type of surgery	Immediate	30 (13.39)	40 (17.86)	0.81	0.367
	Delayed	76 (33.93)	78 (34.82)		
Reason of failure	Smoking	22 (9.82)	10 (4.46)	106.61	0.001*
	Peri-implantitis	42 (18.75)	14 (6.25)		
	Osseointegration	0 (0)	76 (33.93)		
	Diabetes	22 (9.82)	14 (6.25)		
	Bruxism	20 (8.93)	4 (1.79)		
Implant length (mm)	≤10	50 (22.32)	48 (21.43)	7.81	0.099
	11	34 (15.18)	48 (21.43)		
	12	22 (9.82)	22 (9.82)		
Implant diameter (mm)	3.6	14 (6.25)	10 (4.46)	1.79	0.408
	3.8	14 (6.25)	6 (2.68)		
	4	40 (17.86)	46 (20.54)		
	4.2	24 (10.71)	40 (17.86)		
	4.5	14 (6.25)	16 (7.14)		

Furthermore, age was a contributing factor, as those experiencing LIF were, on average, older individuals with a mean age of 39.09±10.37 years (p=0.026). The duration of failure was 16.87±4.6 months for LIF, in contrast to 5.71±1.38 months in EIF. Other variables, including gender, jaw type, site, graft necessity, surgical method, implant length, and implant diameter, did not show any significant correlations (Table 2).

**Table 2:** Descriptive analysis of late implant failure and early implant failure. *P-value <0.05 was considered significant. Data were presented in the form of mean±standard deviation (SD). CI: confidence interval

Parameter		Late failure	Early failure	T-value	p-Value
Age (years)	Mean±SD	39.09±10.37	35.85±11.04	2.25	0.026*
	CI at 95%	L37.10-H41.09	L33.83-H37.86		
Duration of failure (months)	Mean±SD	16.87±4.6	5.71±1.38	25.1	0.001*
	CI at 95%	L15.98-H17.75	L5.46-H5.96		

**Figure 2:**
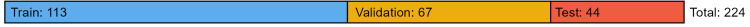
Model for machine learning.

Based on the analysis of decision tree regression utilizing machine learning methodologies to forecast the duration (measured in months) until the failure of dental implants, the following findings were delineated: the principal factors that significantly affect the timeline to implant failure encompassed the length and diameter of the implant, individual smoking behaviors, and systemic conditions such as diabetes mellitus and bruxism. The model underwent training using a dataset comprising 113 samples, with subsequent validation and testing conducted on independent subsets of 67 and 44 samples, respectively (Figure 2).

**Figure 3:**
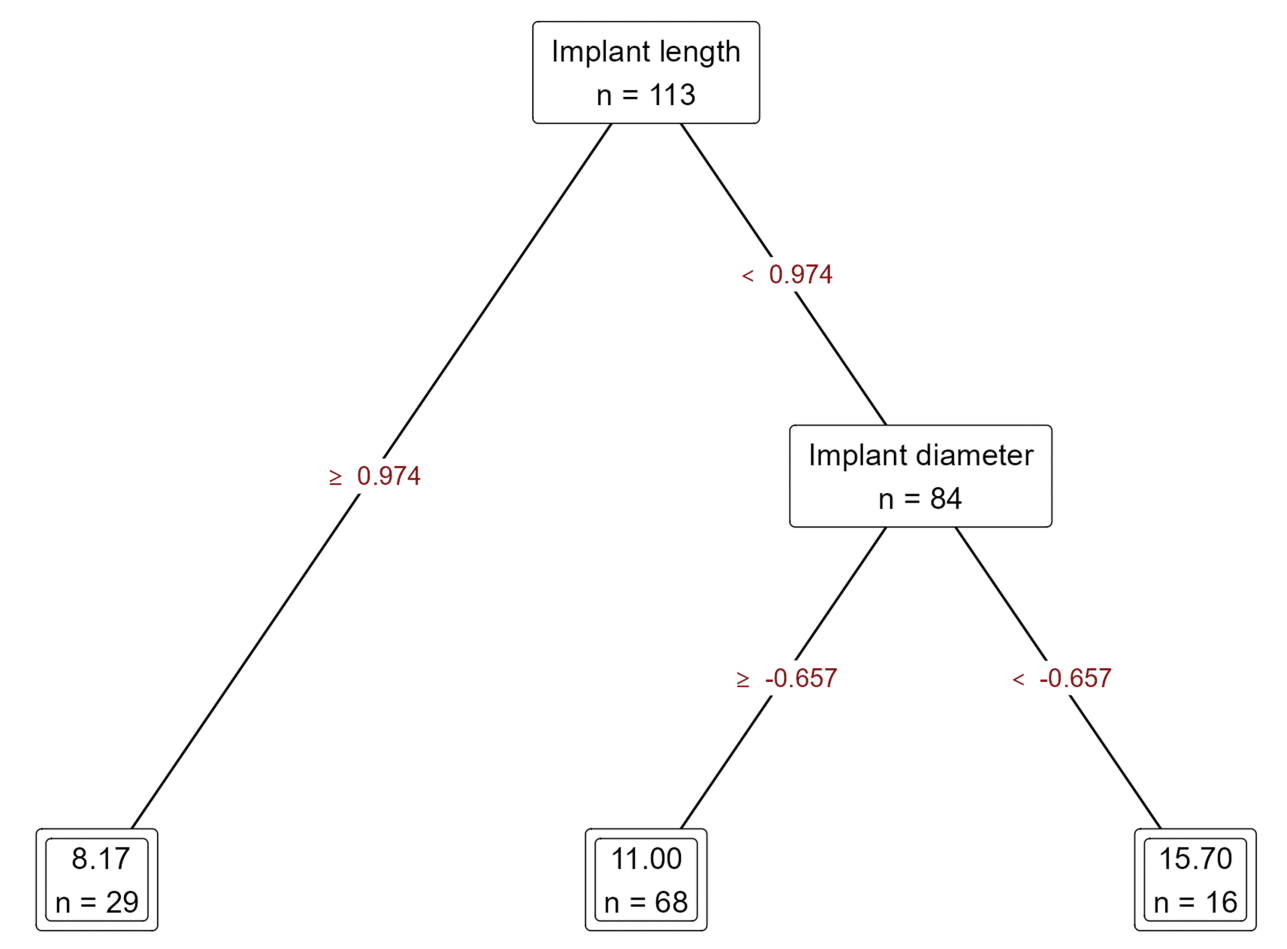
Prediction of duration of implant failure based on implant length and diameter using decision tree regression analysis.

Implant length and diameter are as follows: the length of the implant emerged as the predominant factor with a critical threshold value of 0.974, delineating the distinction between implants with prolonged durability. In the case of implants with shorter lengths (<0.974), the diameter of the implant also affected the duration until failure, with larger diameters (≥ -0.657) correlating with reduced failure intervals (mean ~11 months), whereas smaller diameters (< -0.657) exhibited extended durations (~15.7 months) (Figure 3).

**Figure 4:**
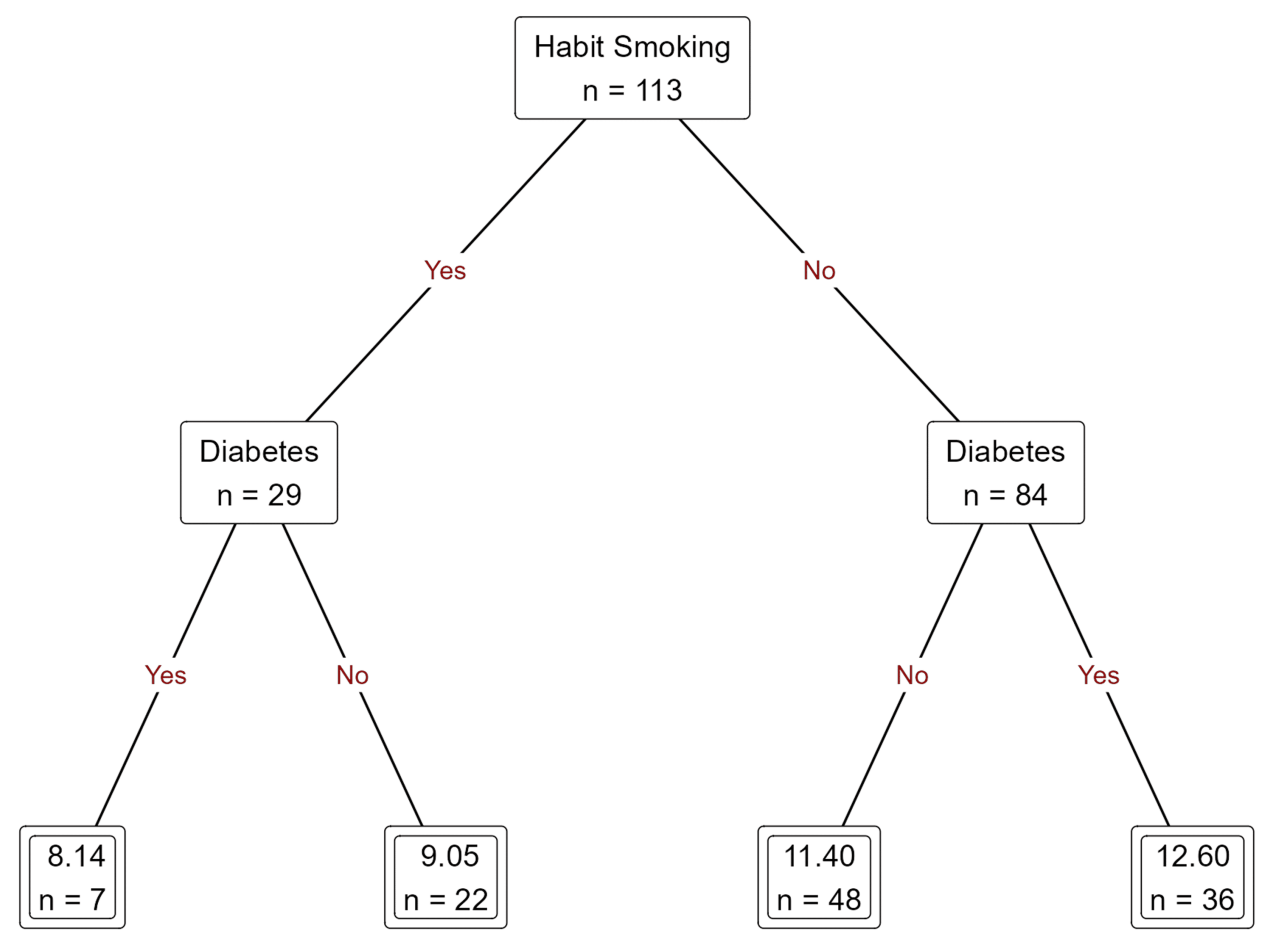
Prediction of duration of implant failure based on habit of smoking in diabetic and non-diabetic patients using decision tree regression analysis.

Systemic and behavioral factors are as follows: smoking has been shown to significantly diminish the longevity of dental implants, particularly in individuals diagnosed with diabetes mellitus. Among smokers, diabetic patients exhibited a notably earlier incidence of implant failure, with a mean duration of approximately 8.14 months, in contrast to their non-diabetic counterparts who smoked and experienced a mean duration of roughly 9.05 months. Furthermore, non-smokers afflicted with diabetes displayed marginally superior implant longevity, averaging around 12.6 months, thereby emphasizing the compounded adverse effects attributable to the interplay between smoking and systemic diseases (Figure 4).

**Figure 5:**
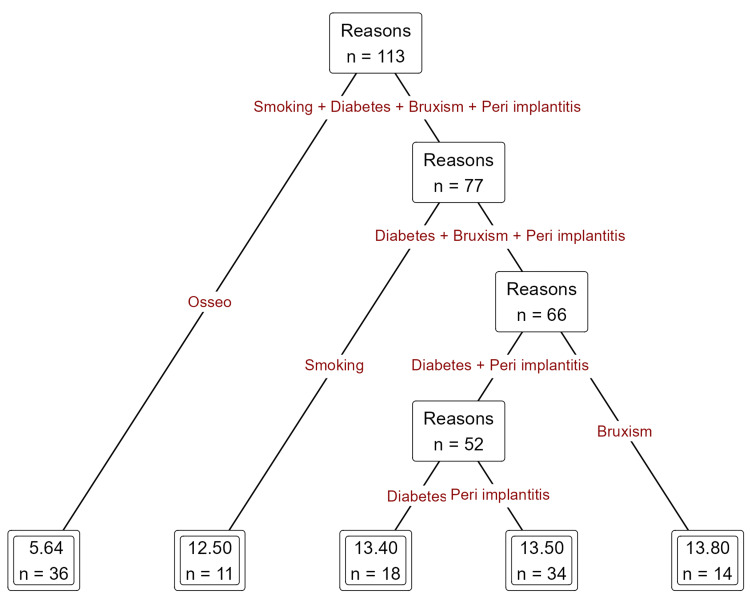
Prediction of duration of implant failure based on reasons of implant failure such as bruxism and peri-implantitis using decision tree regression analysis.

Bruxism and peri-implantitis were correlated with diminished survival duration, especially when compounded by additional risk factors such as diabetes mellitus. Isolated peri-implantitis yielded an average failure duration of approximately 13.4 months, whereas bruxism intensified the failure interval to approximately 13.8 months (Figure 5).

**Figure 6:**
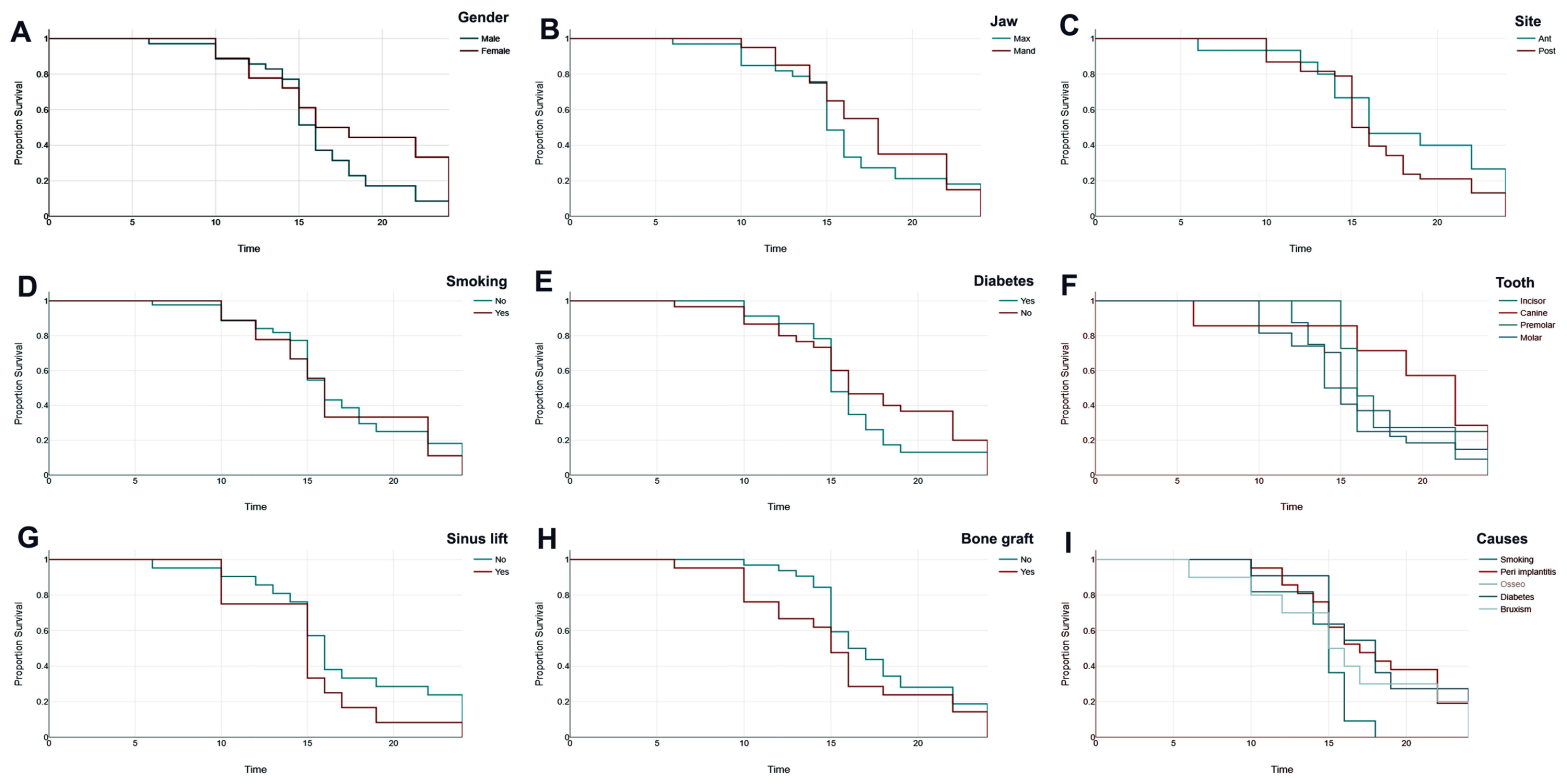
Kaplan-Meier survival analysis for failure of dental implants based on various factors. The images show the effects of the following factors: (A) gender, (B) jaw type, (C) size of implants, (D) smoking, (E) diabetes, (F) tooth type, (G) sinus lift, (H) bone grafting, and (I) causes of failure.

Kaplan-Meier survival analysis elucidates the critical determinants influencing the failure of dental implants. Sex indicated a marginally higher survival rate for females (Figure 6A), whereas maxillary implants demonstrated inferior performance in comparison to mandibular implants (Figure 6B). Posterior implants manifested premature failure relative to anterior implants (Figure 6C). Smoking considerably diminishes implant survival (Figure 6D), as does the presence of diabetes (Figure 6E). Variations in implant survival were noted across tooth types (Figure 6F), with incisors exhibiting the most prolonged survival and molars the least prolonged survival. Procedures such as sinus lifts (Figure 6G) and bone grafts correlated with a slight decrease in survival duration (Figure 6H). Among the identified causes of failure (Figure 6I), peri-implantitis and smoking were the predominant factors, followed by bruxism, diabetes, and complications related to osseointegration (Figures 6A-6I).

## Discussion

Dental implants are routinely used to replace missing teeth. Therefore, the present study was conducted to assess the various risk factors associated with implant failure, particularly EIF and LIF. The results of the present study indicated that EIF was observed in 53% of cases and LIF was observed in 47% of cases. Our results are in agreement with those of Sakka et al. [11]. LIF is typically infrequent and often arises from progressive complications, such as peri-implantitis resulting from inadequate oral hygiene, mechanical breakdown of the implant apparatus, or alterations in the bone density or quality surrounding the implant. These failures usually occur after the implant has successfully integrated, but subsequently succumb to cumulative or progressive influences, whereas EIF occurs mainly due to problems in surgical procedures or bone quality.

The present study indicated that implant failure was more common in males than females. This finding is in agreement with that of Castellanos-Cosano et al. [12]. This could be due to the fact that estrogen in females has a protective effect on bone density and healing, potentially aiding implant osseointegration. Male hormonal profiles do not provide the same benefit and may influence bone metabolism differently, potentially making males more prone to implant complications. Similarly, increased implant failures were observed in older individuals, which is in agreement with a previous study [13].

Shorter implants with smaller diameters are associated with EIF. This could be due to the fact that shorter and narrower implants possess a reduced surface area for interaction with the adjacent bone. This constrained surface area diminishes the likelihood of osseointegration, thereby complicating the ability of the implant to attain stability and support. Smaller implants exhibit diminished stability within the bone, rendering them more vulnerable to movement, which can interfere with osseointegration during the healing phase, resulting in EIF. Implants with a smaller diameter concentrate greater occlusal forces (biting or chewing forces) in a limited area. This amplified stress can exceed the capacity of the implant-bone interface, particularly during the initial stages of healing, thereby increasing the risk of failure. Shorter implants have been associated with mucositis in the mandible [14].

Lack of osseointegration was the main factor for EIF, whereas the sinus lift procedure with grafting and the type of surgical procedure was not significantly associated with implant failure. The lack of osteointegration might be due to various factors such as overheating during implant placement, improper drilling techniques, poor bone quality, infection, and excessive stress. As this was a retrospective study, we could not study the effect of many such factors and identified bruxism as a potential etiological factor for the lack of osseointegration [15]. However, bruxism has also been shown to be a major cause of LIF in previous studies [16,17]. Bruxism is a parafunctional behavior associated with adverse outcomes in dental implants. The primary contributor to failures, including implant fracture, screw loosening, screw breakage, and porcelain fracture, is the excessive occlusal stress experienced by individuals with bruxism [16]. Zupnik et al. conducted a study involving 121 individuals exhibiting bruxism and 220 individuals without bruxism, all of whom collectively possessed 341 dental implants [18]. Their findings indicated the absence of a statistically significant relationship between the occurrence of bruxism and failure rates of dental implants.

Peri-implantitis, smoking, and type 2 diabetes mellitus have also been associated with LIF. Similar risk factors were identified in a systematic review by Do et al. [19]. A previous study identified peri-implantitis as a common risk factor for LIF [20]. Cigarette smoking has been implicated in a multitude of local and systemic health conditions that adversely affect both bone healing and wound recovery. Furthermore, the existing literature substantiates that smoking has a profound impact on early failure [21] and demonstrates a dose-dependent relationship with late failure of dental implants [22]. Consequently, it is imperative for clinicians to exercise prudence and thoroughly educate patients who smoke before proceeding with the implant therapy.

Controlled diabetes is not associated with implant failure [23], whereas uncontrolled diabetes is significantly associated with LIF [24]. Our study revealed that implant failure was more commonly observed in the maxillary jaw and molar teeth, which is in agreement with previous studies [19,25]. Conversely, various investigations conducted by Jemt revealed that the placement of implants within the mandible markedly increased the incidence of late failure [26]. It was further revealed that procedures, such as sinus lifts and bone grafts, correlate with a slight decrease in the survival duration of dental implants. Implants situated in augmented regions or subsequent to sinus elevation procedures may encounter a lag in osseointegration or exhibit incomplete integration in comparison to those implanted in unaltered bones. This temporal delay may elevate susceptibility to micro-movements and instability, potentially compromising the longevity of the implant. Grafting interventions can inflict additional trauma to the adjacent tissues and osseous structures. Over an extended period, the resorption rate of grafted bone may surpass that of natural bone, leading to diminished bone volume surrounding the implant and consequently affecting its stability and long-term durability [27].

Clinical implications

This study underscores the need for careful patient assessment, especially in males, older individuals, smokers, and those with bruxism or uncontrolled diabetes, to minimize the risk of implant failure. Emphasis on maintaining oral hygiene, managing systemic health conditions, and selecting optimal implant dimensions can improve implant longevity and success in clinical practice.

Limitations of the study

The principal constraint of this study was its retrospective design, which consequently hindered our ability to regulate various confounding variables in the analysis, including the category of surgical intervention and orientation of the implants. Moreover, the retrospective design of this study could have led to selection bias. Furthermore, many risk factors such as bone quality, implant angulation, and implant type were not evaluated in this study. Because data are collected post-event, determining the timeline of risk factors and outcomes can be challenging. Therefore, long-term, prospective studies are required.

## Conclusions

Based on the findings of our study, we concluded that smoking considerably diminishes implant survival, as does the presence of diabetes. Procedures such as sinus lifts and bone grafts correlate with a slight decrease in survival duration. Among the identified causes of failure, peri-implantitis and smoking were the predominant factors, followed by bruxism, diabetes, and complications related to osseointegration. Implant failure was more common in the posterior maxillary region in males.
